# Blacklegged ticks, *Ixodes scapularis*, reduce predation risk by eavesdropping on communication signals of *Formica oreas* thatching ants

**DOI:** 10.1098/rsos.231355

**Published:** 2024-01-03

**Authors:** Claire E. Gooding, Charlotte Pinard, Regine Gries, Anand Devireddy, Gerhard Gries

**Affiliations:** Department of Biological Sciences, Simon Fraser University, British Columbia, Canada V5A 1S6

**Keywords:** semiochemicals, predator avoidance, repellent, formic acid, hydrocarbons

## Abstract

Ticks spend most of their life inhabiting leaf litter and detritus where they are protected from sun but preyed upon by ants. Ants secrete chemical communication signals to coordinate group tasks such as nest defence. Ticks that avoid ant semiochemicals—as indicators of ant presence—would reduce predation risk by ants. We tested the hypotheses that: (i) chemical deposits from the thatching ant *Formica oreas* deter blacklegged ticks, *Ixodes scapularis*, (ii) deterrent semiochemicals originate from the ants' poison and/or Dufour's gland(s), and (iii) tick-deterrent semiochemicals serve as alarm-recruitment pheromone components in *F. oreas*. In two-choice olfactometer bioassays, filter paper soiled with ant chemical deposits significantly deterred female and male ticks. Poison and Dufour's gland extracts deterred ticks in combination but not alone. Gas chromatographic-mass spectrometric analyses of gland extracts revealed formic acid as the major constituent in the poison gland and eight hydrocarbons as constituents in the Dufour's gland. Synthetic formic acid and hydrocarbons deterred ticks only when combined. *F. oreas* workers sprayed both formic acid and hydrocarbons when distressed. A synthetic blend of these compounds elicited alarm-recruitment responses by *F. oreas* in behavioural bioassays. All results combined indicate that ticks eavesdrop on the ants' communication system.

## 1. Introduction

Blacklegged ticks, *Ixodes scapularis*, are obligate blood-feeding ectoparasites that feed on mammalian, avian and reptilian hosts [1]. They are most abundant in the eastern and central USA but can be found as far north as the Canadian maritime provinces, and as far south as the Mexican province of Coahuila [2]. Their preferred hosts are white-tailed deer, *Odocoileus virginianus*, and rodents, but they also feed opportunistically on humans [1]. Blacklegged ticks carry 16 known human pathogens including *Borrelia burgdorferi,* the causative agent of Lyme Disease [3]. In the USA alone, there are an estimated 300 000 cases of Lyme Disease annually, making *I. scapularis* a species of significant medical importance [4].

Blacklegged ticks spend most of their life taking refuge in leaf litter and detritus [5]. The high humidity and protection from sun afforded by these microhabitats are essential for the survival of ticks which are prone to desiccation [6,7]. However, ticks share these microhabitats with numerous generalist arthropod predators [8]. Opportunistic predation by ants on leaf litter-dwelling ticks, beetles and spiders [8–10] significantly impacts tick survival and/or distribution [11–13]. For example, the abundance of *Ixodes* ticks is affected by both European fire ants, *Myrmica rubra,* and red wood ants, *Formica polyctena* [12,13], and the *Amblyomma* tick burden on small mammals is reduced in areas inhabited by red imported fire ants, *Solenopsis invicta* [14]. Both ant predation on ticks, and tick avoidance of ant semiochemicals, may underlie the effects of ants on tick abundance and distribution.

Predator-derived cues can prompt predator avoidance behaviours in prey [15] but the type of cue, and its specific characteristics mediating predator avoidance behaviours by prey are often not investigated. Ants prey on many arthropods, including ticks, and often exert both consumptive and non-consumptive effects on prey species [12,13,16]. Ants use a plethora of chemical communication signals to coordinate group tasks such as nest defence, brood care and foraging behaviour [17]. Potential prey of ants, including spiders, bees and fruit flies, eavesdrop on these ant communication signals, and avoid areas where they have been deposited [18–22]. Whether ticks avoid ant semiochemicals has not yet, to our knowledge, been investigated.

**Figure 1.**
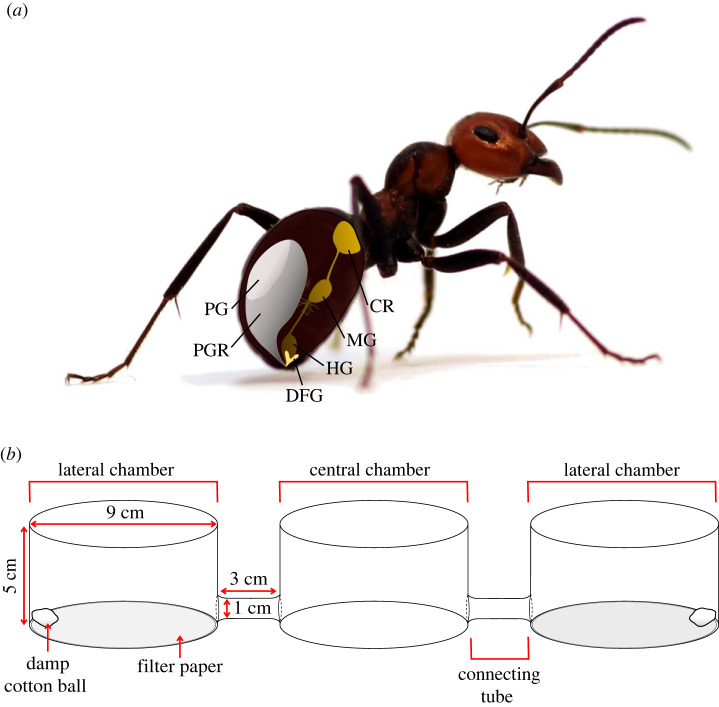
Drawings illustrating (*a*) the location of the poison gland and Dufour's gland in *Formica oreas* worker ants, and (*b*) the olfactometer used in tick bioassays. For bioassays, the lateral chambers of the olfactometer received a piece of filter paper treated with a treatment or control stimulus, and a damp cotton ball to increase relative humidity. A single bioassay tick was released into the central chamber, and was considered a responder, if it was found at the end of the bioassay in a lateral chamber, or in a connecting glass tube closer to a lateral chamber than to the central chamber. PG, poison gland; PGR, poison gland reservoir; DFG, Dufour's gland; CR, crop; MG, midgut; HG, hindgut.

Avoidance of ant semiochemicals—as indicators of ant presence—would be adaptive for ticks, if these semiochemicals were to (i) accumulate in areas inhabited or frequently visited by ants, and (ii) reliably signal the current or imminent presence of any species of predatory ant. Constituents in the ants’ poison and Dufour's glands (for their location see figure 1*a*) have multiple communication functions including alarm-recruitment of nest-mates [23–31], and thus are frequently secreted, and probably accumulate, in areas inhabited by ants. As gland constituents are highly conserved across formicine ants, they could be adopted by prey as generic predator recognition and avoidance cues. Poison and Dufour's gland secretions typically comprise formic acid and assorted hydrocarbons, respectively [23–26,28,29,32–35]. Whereas hydrocarbons may originate from multiple sources other than the Dufour's gland, formic acid is rather indicative of ant presence, and thus could—alone or in combination with specific hydrocarbons—reliably indicate predation risk by ants.

The thatching ant *Formica oreas* is a representative member of the *Formica rufa* species group. It occurs in various woodland and prairie habitats and is known for its conspicuous thatch-mound nests [36]. With some overlap in the geographical distribution of *I. scapularis* and *F. oreas* [36–38], and *I. scapularis* and other woodland-dwelling species in the *Formica rufa* group [39,40], it is conceivable that *I. scapularis* interacts with *F. oreas*, and with other *F. rufa* group ants, in a predator–prey relationship. *Formica oreas* is an aggressive and competent predator of various arthropods [38,41]. It has not yet been documented to prey upon *I. scapularis* or other ticks but its congener *F. polyctena* curtails the abundance of *Ixodes* ticks in Europe [13].

Working with *F. oreas* worker ants and *I. scapularis* ticks, we tested the hypotheses that: (i) ant semiochemical deposits deter ticks, (ii) tick-deterrent semiochemicals originate from the ants' poison and/or Dufour's gland(s), and (iii) the tick-deterrent semiochemicals serve as alarm-recruitment pheromone components in ants.

## 2. Material and methods

### 2.1. Tick maintenance

Adult *I. scapularis* were acquired from BEI Resources (American Type Culture Collection) and the National Tick Research and Education Resource (Oklahoma State University). Groups of 10–12 ticks were housed in 20 ml glass scintillation vials (VWR International, PA, USA) fitted with strips of paper towel as refuge and substrate for climbing, and with a mesh-covered hole (approx. 1 cm) in the lid to enable air exchange. As ticks are prone to desiccation, vials were kept at high relative humidity (85–95%) in a vessel (*d* = 26 cm, *h* = 30 cm) containing a saturated solution of K_2_SO_4_ (99% purity; Alfa Aesar, ON, Canada). To minimize the risk of tick escape, the vessel was retained in a plexiglass box (50 × 35 × 35 cm) which was kept at 22°C and a 14 : 10 light/dark cycle. To prevent mould/fungal growth, vials were washed weekly with Sparkleen (Thermo Fisher Scientific, MA, USA) and dried at 100°C for more than 1 h. Monthly, the vessel was washed and sterilized with Sparkleen and 70% ethanol, respectively, and the K_2_SO_4_ solution was replaced.

### 2.2. Collection and maintenance of ant colonies

Colonies of *F. oreas* were collected in Surrey, British Columbia, Canada (49°10′04.7″ N, 122°41′57.8″ W) in August 2020. Colonies were housed in plastic bins (66 × 40 × 35 cm) filled halfway with nesting material from collection sites. Bins were kept in the Science Research Annex (49°16′33.5″ N, 122°54′55.0″ W) on the Burnaby campus of Simon Fraser University exposed to a 12 : 12 light/dark cycle. Ants were provisioned with mealworms, *Tenebrio molitor,* German cockroaches, *Blattella germanica*, American cockroaches, *Periplaneta americana*, apple slices, and a 20% sugar water solution ad libitum.

### 2.3. General experimental design

Deterrent effects of test stimuli on behavioural responses of ticks were bioassayed in still-air Pyrex glass olfactometers (figure 1*b*), consisting of one central chamber and two lateral chambers (each *d* = 9 cm, *h* = 5 cm), linearly interconnected by glass tubes (*d* = 1 cm, *l* = 3 cm) [42]. Treatment and control stimuli were assigned to the two lateral chambers, alternating the position of stimuli between replicates to account for potential side bias. Both lateral chambers were also fitted with a wet cotton ball (Thermo Fisher Scientific, MA, USA) to ensure sufficiently high humidity. To initiate an experimental replicate, a single tick was introduced into the central chamber, briefly exposed to human exhale to stimulate movement, and then allowed 20 h to respond. A tick was considered a responder, if it was found in a lateral chamber, or in a connecting glass tube closer to a lateral chamber than the central chamber (figure 1). All other ticks were deemed non-responders and excluded from statistical analyses but were reported in figures. To prevent tick escape, all three chambers of the olfactometer were sealed with Parafilm (Bemis, WI, USA) for the duration (20 h) of the experiment. To minimize the potential for tick escape, and to prevent ticks from sensing cues (e.g. convective heat, infrared radiation, CO_2_) originating from experimentalists that initiated or scored experiments, olfactometers (*n* = 10–15) were housed in a plexiglass box (112 × 24 × 14 cm). Experiments were run under a 14 : 10 light/dark cycle, thus enabling ticks to respond to test stimuli while maintaining a circadian rhythm. After each experiment, olfactometers were washed with Sparkleen (Thermo Fisher Scientific), thoroughly rinsed with distilled water, and dried at 100°C for more than 1 h.

### 2.4. Specific experiments

#### 2.4.1. Hypothesis 1: ant semiochemical deposits deter ticks

##### 2.4.1.1. Collection, and behavioural effects, of chemical deposits from worker ants

Both lateral chambers of olfactometers were fitted with a piece of filter paper (*d* = 90 mm; Cytiva, MA, USA). To collect chemical deposits of worker ants, the glass tube connecting the randomly assigned lateral treatment chamber to the central chamber was blocked with a damp cotton ball, and 20 cold-anaesthetized (−15°C for 5 min) ants were introduced into the treatment chamber, which was then sealed with parafilm and covered with a Petri dish lid, as was the control chamber. After the ants had roamed 16 h in the treatment chamber, both the treatment and the control chamber were ‘unsealed’, and the ants were allowed to leave the treatment chamber on their own accord, thus minimizing agitation. Then, the cotton ball block was removed from the connecting tube, a tick was introduced into the central chamber, the olfactometer was sealed with parafilm, and the bioassay replicate was initiated. Ant-soiled filter paper (see above) was tested for avoidance responses of male ticks (experiment 1, *n* = 40) and female ticks (experiment 2, *n* = 40).

##### 2.4.1.2. Extraction of ant chemical deposits from filter paper

Filter paper previously cut into squares (2.5–5 mm) and exposed to ants for 16 h (see experiments 1 and 2) was placed into 20 ml scintillation vials for extraction of ant chemical deposits. Each of four scintillation vials received filter paper squares originating from three 90 mm wide filter paper discs. To extract both polar and non-polar compounds, filter papers were sequentially extracted in hexane (7 ml per vial × 4 vials) and dichloromethane (DCM; 7 ml per vial × 4 vials), each for 20 min at room temperature. Following extractions, hexane and DCM extracts were pipetted into separate clean 20 ml scintillation vials. Prior to analyses, combined hexane extracts and combined DCM extracts were concentrated to 12 ml each under a nitrogen stream.

#### 2.4.2. Hypothesis 2: deterrent semiochemicals originate from the ants' poison and/or Dufour's gland(s)

##### 2.4.2.1. Extractions of poison and Dufour's glands

**Figure 2.**
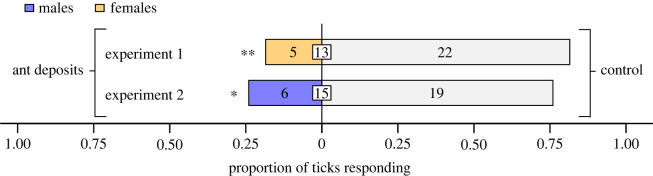
Proportion of female and male blacklegged ticks, *Ixodes scapularis*, responding in olfactometers (figure 1*b*) to filter paper previously soiled, or not (control), with chemical deposits of 20 *Formica oreas* worker ants. Numbers in bars represent the total number of ticks choosing a stimulus, and numbers in white inset boxes represent the total number of non-responding ticks. Asterisks indicate significant avoidance of filter paper with ant chemical deposits (exact binomial tests; *p < 0.05, **p < 0.01).

Worker ants were collected from laboratory colonies (see above) and cold-euthanized in a −15°C freezer, where they remained until dissection (up to 4 days). Ants were dissected in chilled, distilled water under a dissecting microscope (ZEISS Stemi 2000), using fine-tipped forceps (Almedic, FR, CH) and insect pins. In total, 310 poison glands (with reservoirs) and 315 Dufour's glands were excised and placed in separate 4 ml glass vials (VWR International, PA, USA) each containing DCM (1 ml). To minimize passive emanation of gland constituents from open vials during dissections, vials were kept on ice. To facilitate gland extractions, both samples were first vortexed for 60 s to homogenize gland tissues and then kept for 15 min at room temperature. Following extractions, samples were filtered through glass wool into clean 4 ml glass vials capped with Teflon-lined lids. To minimize cross-contamination between poison gland and Dufour's gland constituents, all tools were cleaned with DCM between gland excisions, and ruptured glands were omitted. Both filter paper extract and gland extracts were analysed to determine the origin of chemical constituents in filter paper extract that proved deterrent to ticks in experiments 1 and 2 (see Results; figure 2).

##### 2.4.2.2. Behavioural effects of poison and Dufour's gland contents

Parallel experiments 3–8 (*n* = 40 each) tested avoidance behaviour of male and female ticks in response to: poison gland extract (experiments 3 and 4), Dufour's gland extract (experiments 5 and 6), and both combined (experiments 7 and 8), all versus a solvent control. Treatment stimuli were presented at one gland equivalent (table 1) dissolved in 500 µl of DCM, whereas an equal volume of DCM was used as the control stimulus. Stimuli were applied dropwise evenly spread across the 90 mm wide filter paper disc. After 5 min of DCM evaporation, a tick was placed into the central chamber of the olfactometer, and the bioassay was initiated.

**Table 1.** Mean amount (ng) of chemical constituents quantified in poison and Dufour's gland extracts of *Formica oreas* workers.

compound	ng per gland equivalent	gland
formic acid	10 000	poison
undecane	60 000	Dufour's
tridecane	5100	Dufour's
(*Z*)-4-tridecene	3600	Dufour's
heptadecane	1140	Dufour's
(*Z*)-9-tricosene	900	Dufour's
pentadecane	840	Dufour's
(*Z*)-9-nonadecene	840	Dufour's
(*Z*)-9-heneicosene	240	Dufour's

With experimental data demonstrating that poison gland extract and Dufour's gland extract in combination elicit avoidance responses by ticks (see Results, figure 4), experiments 9–14 tested avoidance behaviour of ticks in response to synthetic equivalents of compounds present in: poison gland extract (experiments 9 and 10), Dufour's gland extract (experiments 11 and 12), and both combined (experiments 13 and 14). Like gland extracts, synthetic blends were tested at one gland equivalent, and were applied in 500 µl DCM, with the same volume of DCM serving as the control stimulus.

#### 2.4.3. Hypothesis 3: the tick-deterrent semiochemicals serve as alarm-recruitment pheromone components in ants

##### 2.4.3.1. Collection of *Formica oreas* defensive sprays

To elicit defensive behaviour by ants, a pair of fine-tipped forceps was inserted vertically into the nesting box with the tips touching the substrate. Once a single ant had bitten onto the forceps indicating defensive behaviour, the forceps—together with the ant hanging on them—were withdrawn, and a piece of filter paper (5 × 20 mm) was placed for 10 s under the ant's abdominal tip to capture her defensive spray(s). Then, the filter paper was extracted sequentially in hexane (500 µl) and DCM (500 µl) for 60 s each. This process was repeated with 20 ants randomly selected from three laboratory colonies. Prior to analyses of samples, they were concentrated to 100 µl under a nitrogen stream.

##### 2.4.3.2. Ant recruitment to micro-locations treated with poison and Dufour's gland semiochemicals

To test alarm-recruitment behaviour of *F. oreas* worker ants in response to poison and Dufour's gland semiochemicals, we followed an established protocol [43]. For each bioassay replicate (*n* = 20), two filter paper discs (90 mm each) were placed in a plexiglass bioassay arena (64 × 44 × 10 cm) 41 cm apart. By random assignment, one filter paper was treated with a synthetic blend of formic acid and hydrocarbons dissolved in DCM (10 µl) at one gland equivalent (table 1), whereas the control disc received only DCM (10 µl). To initiate a bioassay, a 15 ml Falcon tube (Thermo Fisher Scientific, Waltham, MA, USA) containing five ants was placed into the arena such that the tube's tapered tip was flush with the arena floor and equidistant to the two filter paper discs. Then, the cotton plug was removed from the 0.7 cm diameter hole cut in the tube's tip, thus allowing the ants to enter the arena on their own accord. Once the first ant had entered the arena, the ants' behaviour was filmed (Canon EOS Rebel T7, Canon, Tokyo, Japan) for 150 s. Videos were reviewed using VLC Media Player (version 3.0.17.4), and visits to each of the two filter paper discs were counted. Multiple visits were counted for a single ant, if she had completely left the filter paper disc between visits.

##### 2.4.3.3. Chemical analyses of ant-soiled filter paper, gland extracts and defensive sprays

Aliquots (2 µl) of filter paper extracts in hexane and DCM were analysed in splitless mode (purge valve open for 0.8 min) by gas chromatography-mass spectrometry (GC-MS), using an Agilent 7890B gas chromatograph (GC) fitted with a DB-5 GC-MS column (30 m × 0.25 mm internal diameter, film thickness 0.25 µm), and coupled to a 5977A mass selective detector. The GC injector port was set to 250°C, the MS source to 230°C and the MS quadrupole to 150°C. With helium as the carrier gas (flow rate: 35 cm s^−1^), the following temperature program was used: 40°C held for 5 min, 10°C min^−1^ to 280°C (held for 10 min). Compounds in extracts were identified by comparing their retention indices [44] and mass spectra with those of authentic standards that were purchased or synthesized. Double bond positions in unsaturated hydrocarbons were determined by treating aliquots of extracts with dimethyl disulfide (DMDS) [45], and by analysing DMDS derivatives for double bond positions. To test for the presence of formic acid which chromatographs poorly and thus is easily missed or incorrectly quantified, further aliquots of extracts were treated with 1-decanol to derivatize formic acid to decyl formate which readily chromatographs [46].

Poison and Dufour's gland extracts were analysed using the same protocol. However, because derivatization of formic acid to decyl formate enabled detection, but not accurate quantification, of formic acid in poison gland extract, formic acid was quantified instead using a 7964 Agilent Headspace Sampler coupled to a Varian 2000 Ion Trap GC-MS fitted with a DB-FATWAX Ultra Inert GC column (30 m × 0.25 mm internal diameter). To this end, we applied one gland equivalent of poison gland extract to filter paper (Cytiva, MA, USA) in a 20 ml vial, which was then sealed with a 20 mm outer diameter silicon septum and a crimped cap. The vial was heated to 150°C, and headspace volatiles were withdrawn with an automated syringe and subjected to GC-MS analysis, using the following temperature programme: 40°C (10 min), 10°C min^−1^ until 200°C.

Defensive sprays were analysed using the same protocol as described for the filter paper extracts.

##### 2.4.3.4. Purchase and synthesis of semiochemicals

Undecane, tridecane, heptadecane, (Z)-9-tricosene, pentadecane and (*Z*)-9-heneicosene were purchased from Sigma-Aldrich (St Louis, MO, USA). Formic acid was purchased from Anachemia Science (Rouses Point, NY, USA). (Z)-4-tridecene and (Z)-9-nonadecene were synthesized in our laboratory as previously described [47].

### 2.5. Statistical analysis

All data were analysed using R-studio (version 2022.07.2 + 576), and all figures were prepared using R-studio and Inkscape (version 0.92.4). The glmmTMB [48], DHARMa [49] and ggeffects [50] packages were used to aid in analyses, and the scales package [51] was used to aid in creating figures. When we tested for potential side bias in pre-screening experiments, ticks—in the absence of any test stimuli—chose equally often either one of the two lateral chambers. Tick avoidance behaviour in response to treatment stimuli was then assessed by comparing the ratio of treatment and control responses to a hypothetical response ratio of 1 : 1, using a two-sided exact binomial test and excluding non-responders from analyses. This statistical approach aligns with the best practises for analysis of data collected in dual-choice olfactometer bioassays [52]. A Cohen's *g* test was used to calculate and categorize effect sizes as ‘negligible’, ‘small’, ‘medium’ or ‘large’ based on guidelines established by Cohen [53]. Ant attraction to filter paper discs treated with synthetic ant semiochemicals was modelled using a zero-inflated generalized linear mixed model with a Poisson distribution, using treatment as a fixed effect and replicate as a random effect. We assessed the effect of treatment using a likelihood ratio test.

## 3. Results

### 3.1. Hypothesis 1: ant semiochemical deposits deter ticks

#### 3.1.1. Behavioural effects of chemical deposits from worker ants

**Figure 3.**
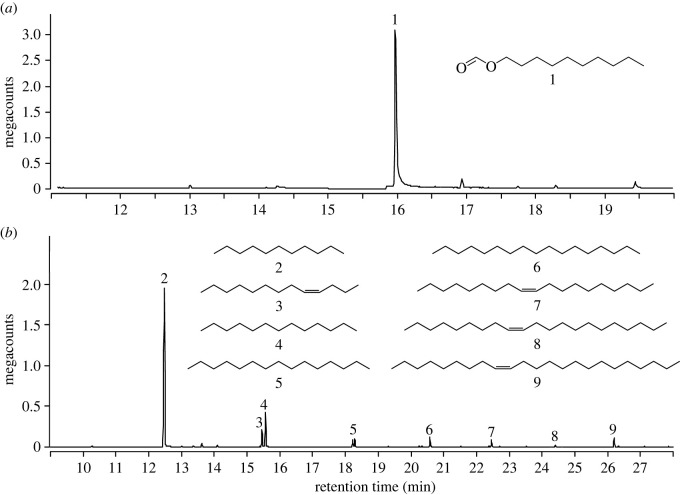
Total ion chromatograms of poison gland extract (*a*) and Dufour's gland extract (*b*) obtained from worker ants of *Formica oreas*. The poison gland extract was treated with 1-decanol to derivatize formic acid (which chromatographs poorly) to decyl formate (1). Constituents of the Dufour's gland are undecane (2), (Z)-4-tridecene (3), tridecane (4), pentadecane (5), heptadecane (6), (Z)-9-nonadecene (7), (Z)-9-heneicosene (8) and (Z)-9-tricosene (9). Chromatography: DB-5 column; temperature programme: 40°C (5 min), 10°C min^−1^ to 280°C (held for 10 min).

When ticks in olfactometers (figure 1*b*) were offered a choice between filter paper with or without ant chemical deposits, female and male ticks were significantly deterred by filter paper soiled with ant chemical deposits (exact binomial tests; experiment 1: females, *n* = 27, *p* = 0.0015; experiment 2: males, *n* = 25, *p* = 0.015; figure 3). There was a ‘large’ effect size for avoidance responses by females (Cohen's *g*: 0.26) and males (Cohen's *g*: 0.31).

#### 3.1.2. Identification of ant chemical deposits in filter paper extracts

GC-MS analyses of filter paper extracts in DCM and hexane, before and after chemical derivatization, revealed the presence of formic acid and hydrocarbons, respectively. The hydrocarbons consisted of undecane, tridecane, pentadecane, heptadecane, (*Z*)-4-tridecene, (*Z*)-9-tricosene, (*Z*)-9-nonadecene and (*Z*)-9-heneicosene.

### 3.2. Hypothesis 2: deterrent semiochemicals originate from the ants' poison and/or Dufour's gland(s)

**Figure 4.**
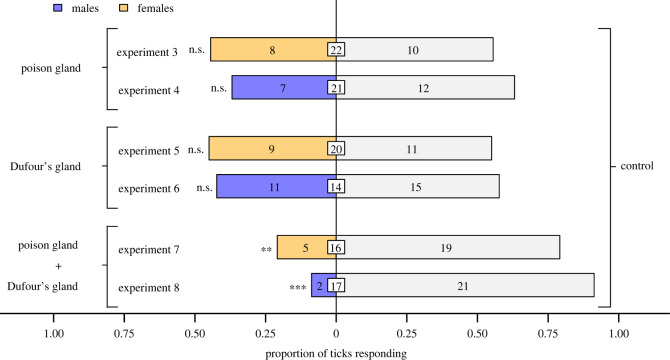
Proportion of female and male blacklegged ticks, *Ixodes scapularis,* responding in olfactometers (figure 1*b*) to extracts of the poison gland (experiments 3 and 4), Dufour's gland (experiments 5 and 6) or to both extracts combined (experiments 7 and 8), all obtained from worker ants of *Formica oreas* and tested at a dose of one gland equivalent. Numbers in bars represent the total number of ticks choosing a stimulus, and numbers in white inset boxes represent the total number of non-responding ticks. Asterisks indicate significant avoidance of the treatment stimulus (exact binomial tests; **p < 0.01, ***p < 0.001; n.s., not significant).

In two-choice olfactometers (figure 1*b*), ticks were deterred neither by poison gland extract (exact binomial tests; experiment 3, females, *n* = 18, *p* = 0.81; experiment 4, males: *n* = 19, *p* = 0.36) nor by Dufour's gland extract (exact binomial tests; experiment 5: females, *n* = 20, *p* = 0.82; experiment 6: males, *n* = 26, *p* = 0.56; figure 4). However, ticks were significantly deterred by extract of both the poison gland and the Dufour's gland (binomial test; experiment 7: females, *n* = 24, *p* = 0.0066; experiment 8: males, *n* = 23, *p* = 6.6 × 10^−5^). There was a ‘large’ effect size for avoidance responses by females (Cohen's *g* = 0.29) and males (Cohen's *g* = 0.41) to combined gland extracts.

#### 3.2.1. Identification of compounds in gland extracts

GC-MS analyses of gland extracts in DCM, before and after chemical derivatization, revealed the presence of formic acid in poison gland extracts and of hydrocarbons in Dufour's gland extracts (figure 2). Of all compounds detected, formic acid was most abundant followed—in order of decreasing abundance—by undecane, tridecane, (Z)-4-tridecene, heptadecane, (Z)-9-tricosene, pentadecane, (Z)-9-nonadecene, (Z)-9-heneicosene (table 1).

#### 3.2.2. Behavioural responses of ticks to synthetic poison and Dufour's gland constituents

**Figure 5.**
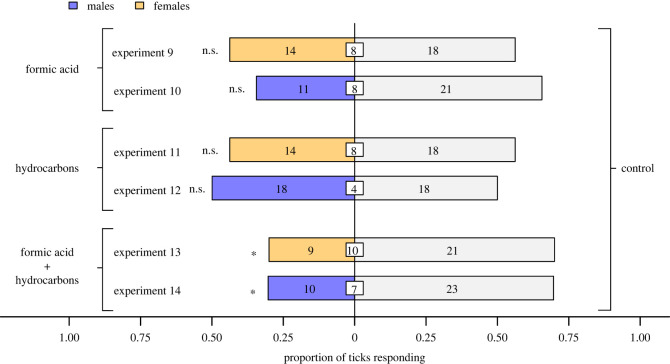
Proportion of female and male blacklegged ticks, *Ixodes scapularis,* responding in olfactometers (figure 1*b*) to synthetic compounds identified in the poison gland (formic acid), and in the Dufour's gland (various hydrocarbons; figure 2) of *Formica oreas* worker ants. All compounds were tested at a dose of one gland equivalent (table 1). Numbers in bars represent the total number of ticks choosing a stimulus, and numbers in white inset boxes represent the total number of responding ticks. Asterisks (*) indicate significant avoidance of the treatment stimulus (exact binomial tests; *p < 0.05; n.s., not significant).

In two-choice olfactometer bioassays (figure 1*b*), ticks were deterred neither by formic acid (poison gland constituent) (exact binomial tests; experiment 9: females, *n* = 32, *p* = 0.60; experiment 10: males, *n* = 32, *p* = 0.11; figure 5) nor by hydrocarbons (Dufour's gland constituents) (exact binomial tests; experiment 11: females: *n* = 32, *p* = 0.60; experiment 12, males, *n* = 36, *p* = 1.0; figure 5). However, formic acid and hydrocarbons in binary combination deterred ticks (exact binomial tests; experiment 13: females, *n* = 30, *p* = 0.043; experiment 14: males, *n* = 33, *p* = 0.035). There was a ‘medium’ effect size for the avoidance responses by females (Cohen's *g* = 0.20) and males (Cohen's *g* = 0.20) to formic acid and hydrocarbons in combination.

### 3.3. Hypothesis 3: tick-deterrent semiochemicals serve as alarm-recruitment pheromone components in ants

#### 3.3.1. Identification of compounds in defensive sprays

GC-MS analyses of filter paper sprayed upon by distressed single workers of *F. oreas* revealed formic acid and all the hydrocarbons identified in the poison and Dufour's gland extract, respectively (table 1), indicating that the ants discharged the content of both glands in their defensive sprays that were experimentally provoked.

#### 3.3.2. Ant recruitment to micro-locations treated with synthetic poison and Dufour's gland semiochemicals

**Figure 6.**
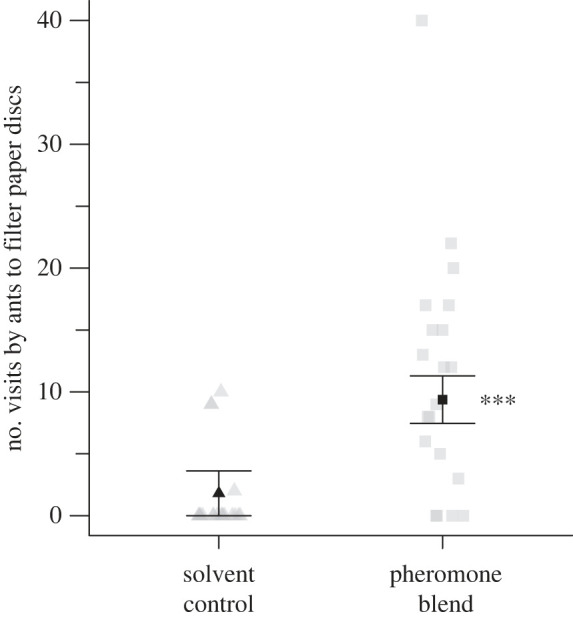
Number of visits by *Formica oreas* worker ants, tested in groups of five (*n* = 20), to paired filter paper discs placed 41 cm apart in a bioassay arena (64 × 44 × 10 cm), and treated—at one ant equivalent—with either a synthetic pheromone blend of poison and Dufour's gland components dissolved in dichloromethane (DCM) or a DCM solvent control. Grey symbols show the number of visits in each replicate and black symbols the estimated marginal means (± 95% confidence interval). Asterisks (***) indicate significantly more visits to the disk treated with synthetic alarm-recruitment pheromone (likelihood ratio test; *p* < 0.001).

Synthetic blends of poison and Dufour's gland pheromone components elicited alarm-recruitment responses by *F. oreas* workers in arena bioassays (figure 6). Workers visited micro-locations treated with formic acid and hydrocarbons significantly more often than micro-locations treated with a solvent control (likelihood ratio test; $\chi_{1}^{2}=40.5$, *p* = 1.6 × 10^−9^).

## 4. Discussion

Our findings support the hypotheses that: (i) chemical deposits of *F. oreas* worker ants deter *I. scapularis* ticks, (ii) the deterrent semiochemicals originate from the ants' poison and Dufour's glands, and (iii) the tick-deterrent semiochemicals serve as alarm-recruitment pheromone components of *F. oreas* workers. Combined, the formic acid that ants discharge from their poison glands, and the various hydrocarbons that ants discharge from their Dufour's gland, produce the pheromone blend that attracts nest-mates and deters ticks.

The deterrence of ticks in bioassays was contingent upon the presence of both poison and Dufour's gland extracts, or their respective constituents (figures 4 and 5). Expectedly, both poison and Dufour's gland constituents were present in defensive sprays of *F. oreas* workers, indicating that distressed ants discharge the content of both glands to alarm and recruit nest-mates, and that *I. scapularis* ticks eavesdrop on the ants' complete array of alarm-recruitment communication signals.

Synergism between alarm-recruitment pheromone components from the poison and the Dufour's gland is common in formicine ants. This type of synergism was first noted in the carpenter ant *Camponotus pennsylvanicus,* where distressed workers spray formic acid together with *n*-undecane. In *C. pennsylvanicus*, formic acid stimulates frenzied running behaviour in nest-mates and, even by itself, attracts them, but its attractiveness is greatly increased in combination with *n*-undecane which is mildly attractive alone [54]. Similarly, workers of western carpenter ants, *Camponotus modoc*, convey distress using two acids (formic, benzoic) and a set of alkanes from poison and Dufour's glands, respectively [43]. Formic acid was the most abundant constituent in the poison gland of *F. oreas* workers, as was undecane in the ants' Dufour's gland (figure 2). The same chemical constituents are released from poison and Dufour's glands of other formicine ants [23–26,28,29,32–35], suggesting that ticks could exploit them as generic cues indicative of predation risk by ants. That the synthetic blend of formic acid and alkanes/alkenes in this study was not as deterrent to ticks as combined extracts of the poison and Dufour's gland is attributed to contrasting release dynamics of natural and synthetic compounds, rather than missing pheromone components in the synthetic blend. We predict that exocrine gland secretions contain constituents that slow the release of volatile pheromone components, such as formic acid, comparable perhaps to the role of major urinary proteins in urine deposits of rodents that facilitate sustained release of volatile sex pheromone components [55].

Poison and Dufour's gland secretions of ants serve both communicative and sanitary functions. In formicine ants, formic acid from the poison gland and hydrocarbons from the Dufour's gland alarm and recruit nest-mates [23–29,35,56]. Moreover, workers of *Formica paralugubrius* spray formic acid—probably in combination with the Dufour's gland content—as a potent disinfectant on their nesting material [30], as do workers of the leaf-cutting ant *Acromyrmex subterraneus subterraneus* and the Southeast Asian weaver ant *Polyrhachis dives* [31]. Formic acid is effective against *Metarhizium*, a common fungal pathogen of ants [30,57]. The distinctively acidic smell of *F. oreas* nest mounts (C. E. Gooding 2021, personal observation)—even in the absence of any defensive behaviour—again implies the use of formic acid as a disinfectant, but this inference has still to be experimentally tested. While ants spray formic acid to disinfect their nesting material, ticks may eavesdrop on these disinfectant sprays to evade ant predation. Nest mounds and their immediate surroundings would have the highest concentration of formic acid and Dufour's gland hydrocarbons, and thus would signal severe predation risk. This concept would explain why the volume of *F. polyctena* nesting material was inversely correlated with tick abundance near nests [13]. Avoiding areas with significant formic acid smell would help reduce ant predation risk and thus be adaptive to *I. scapularis.* Even if some formicine ants were to use formic acid and Dufour-gland hydrocarbons only for communicative functions, their frequent use may still result in high concentrations near nests. Currently, *I. scapularis* is thought to engage in minimal (less than 1 m) lateral off-host movement [58], but how far ticks may move in response to ant predatory cues to lower ant predation risk has not yet been studied in field experiments.

As ticks eavesdrop on pheromonal signals released from the poison and Dufour's glands of *F. oreas*, they may conceivably eavesdrop also on ant communication signals originating from other exocrine glands such as mandibular glands. In *F. oreas*, the functional role of mandibular gland constituents is not known but mandibular gland constituents of other ants play roles in the context of mating or alarm signalling [59,60]. The chemical composition of mandibular glands is complex, including compounds such as citronellol, citronellal, *cis*-citral, limonene, cymen, methyl salicylate and geranial [26,61,62]. Citronellol and citronellal in mandibular glands of *Lasius umbratus* elicit alarm and defensive behaviour in nest-mates [58]. Serving as mandibular gland pheromone components of ants, these components—like poison and Dufour-gland pheromone components—could be deterrent to ticks, because some of these compounds occur in plant essential oils which are repellent to ticks [63–65]. The deterrence of plant essential oils to ticks has often been credited to their strong odour or potential toxicity at high concentration [63] but, instead, may be owing to the presence of constituents also in ant exocrine glands. Regardless, it would be interesting to test mandibular gland constituents for tick deterrence and potential synergism between poison, Dufour's and mandibular gland constituents.

In conclusion, we show that poison and Dufour's gland constituents of *F. oreas* worker ants synergistically deter female and male *I. scapularis* ticks, indicating that ticks eavesdrop on the ants' alarm communication signals. A synthetic blend of the glands’ constituents—possibly in combination with other tick deterrents such as plant essential oils [65]—could be considered for development as (i) topical tick repellents directly applied to skin, (ii) tick repellents in clothing, and (iii) off-host tick repellents applied to areas highly frequented by humans. Woodchips are already applied along hiking trails to discourage ticks from questing on or near these trails, and could potentially be improved with the addition of deterrent ant semiochemicals [66]. Concern that formic acid—because of its acidic properties—is a dermal or ocular irritant can be dispelled because formic acid, properly formulated at low concentration, is already safely used in cosmetic products [67]. If synthetic ant semiochemicals were to be developed as tick deterrents for human protection, their deterrent effect would need to be further tested in the presence of host cues that attract foraging ticks, e.g. deer-associated cues [68,69].

## Data Availability

Data and code are accessible at Dryad: https://doi.org/10.5061/dryad.fbg79cp21 [70].
